# In Global Health Research, Is It Legitimate To Stop Clinical Trials Early on Account of Their Opportunity Costs?

**DOI:** 10.1371/journal.pmed.1000071

**Published:** 2009-06-09

**Authors:** James V. Lavery, Peter A. Singer, Renee Ridzon, Jerome A. Singh, Arthur S. Slutsky, Joseph J. Anisko, David Buchanan

**Affiliations:** 1 Centre for Research on Inner City Health and Centre for Global Health Research, The Keenan Research Centre in the Li Ka Shing Knowledge Institute of St. Michael's Hospital, Toronto, Ontario, Canada; 2 Dalla Lana School of Public Health and Joint Centre for Bioethics, University of Toronto, Toronto, Ontario, Canada; 3 McLaughlin-Rotman Centre for Global Health, University Health Network and University of Toronto, Toronto, Ontario, Canada; 4 HIV, TB & Reproductive Health Program, Bill & Melinda Gates Foundation, Seattle, Washington, United States of America; 5 Centre for the AIDS Programme of Research in South Africa (CAPRISA), Durban, KwaZulu-Natal, South Africa; 6 Departments of Medicine and Critical Care, and The Keenan Research Centre in the Li Ka Shing Knowledge2 Institute of St. Michael's Hospital, Toronto, Ontario, Canada; 7 Departments of Medicine, Surgery and Biomedical Engineering, University of Toronto, Toronto, Ontario, Canada; 8 Independent Consultant, West Chester, Pennsylvania, United States of America; 9 School of Public Health and Health Sciences, University of Massachusetts, Amherst, Massachusetts, United States of America

## Abstract

After the failure of three large clinical trials of vaginal microbicides, a *Nature* editorial stated that the microbicide field “requires a mechanism to help it make rational choices about the best candidates to move through trials” [**1**]. In this month's debate, James Lavery and colleagues propose a new mechanism, based on stopping trials early for “opportunity costs.” They argue that microbicide trial sites could have been saturated with trials of scientifically less advanced products, while newer, and potentially more promising, products were being developed. They propose a mechanism to reallocate resources invested in existing trials of older products that might be better invested in more scientifically advanced products that are awaiting clinical testing. But David Buchanan argues that the early stopping of trials for such opportunity costs would face insurmountable practical barriers, and would risk causing harm to the participants in the trial that was stopped.

## Abstract 2

This debate examines the ethics of reallocating resources invested in existing trials of older products into new trials of more scientifically advanced products.

## James Lavery and Colleagues' Viewpoint: We Should Be Able To Reallocate Clinical Trial Resources to Scientifically More Promising Technologies

An intravaginal microbicide that can block the transmission of HIV remains elusive in the wake of a long, agonizing series of scientific setbacks and social challenges [2],[3]. Clinical trials require a large amount of financing, research and clinical infrastructure, investigator expertise and time, and goodwill and buy-in of communities from which thousands of research participants are enrolled for many years. The extent to which these resources can continue to be mobilized to meet the anticipated demand in HIV prevention trials, and other areas of global health research, is unknown.

We turned our attention to the microbicide field in October 2006. At that time, available clinical trial sites in the developing world were nearing saturation with trials of scientifically less advanced, less promising, “first generation” products (e.g., polyanion, surfactant, and buffering microbicides), while newer, potentially more promising, “next generation” antiretroviral-containing products were being developed, some of which were nearly ready for phase III clinical trials [4]. There was growing concern that the “cycle time” of development of next generation products would outpace the capacity for clinical trial testing, resulting in a queue for testing of antiretroviral-containing microbicides. This scenario did not come to fruition as unexpected product failures and prematurely halted trials emptied several large clinical trial sites and resolved the “microbicides queue problem,” albeit in an extremely disappointing fashion. But a *Nature* editorial at the time called for a mechanism to address this problem [1].

Although the microbicides queue problem receded, it would be unwise to view it as an isolated case. With massive investments in discovery and development in global health during the past decade, promising new drugs, vaccines, and devices could emerge ready for phase III testing more rapidly than the appropriate clinical trial sites can be identified and developed. For example, after decades of dormancy, the prospect of an effective malaria vaccine has been spurred recently by several successful clinical trials [5],[6], a number of promising concepts [7],[8], and a massive commitment of funding, both for research [9] and for advance purchasing of an effective vaccine [10]. The best time to discuss and solve a potential “cycle time” problem is now; not when we are in the throes of a crisis.

### Stopping Early for Opportunity Costs—A Potential Solution to the Cycle Time Problem

One solution to the “cycle time” problem is to propose a new reason for stopping trials early—because of their opportunity costs. Sponsors should have a mechanism to reallocate resources invested in existing trials of older products that might be better invested in more scientifically advanced and promising products that are awaiting clinical testing.

There have been cases of industry-sponsored clinical trials being stopped early. These may be examples of stopping early for opportunity costs where those costs are measured in likelihood of *financial returns* [11]. Here, we are talking instead about opportunity costs measured in *public health benefits* alone.

Early stopping of clinical trials is currently accepted for reasons of safety, efficacy, or futility on the recommendations of data safety and monitoring boards (DSMBs). However, to the best of our knowledge, ours is the first proposal to consider early stopping for public health–related opportunity costs. We believe that under specific conditions, with specified processes and safeguards, opportunity costs may be a legitimate reason for stopping trials early. Our proposal could increase the efficiency and speed of clinical research for the world's most pressing global health problems.

### Standards for Early Stopping Based on Opportunity Costs

The substantive standard for decisions to stop an existing trial in favor of a strategic move to another product should be that a trial of the new product is ready to begin and that there is a compelling scientific rationale for why the new intervention may be considered more promising. Of course, determining when a new product or technology is more promising than one currently undergoing testing requires expert consensus, and there are inherent uncertainties involved.

Up-front commitments must be made to ensure that any early stopping of a trial does not diminish the standard of care provided to individuals enrolled under the initial enrollment agreement. Such commitments may involve preparedness planning, clear communication on the consent form [12] and in the consent process, and prior agreements among the study sponsors and participating communities.

### Opportunity Costs and Priority Setting: The Need for a Legitimate Decision-Making Process

Although there are many bodies associated with clinical trials that have the authority to make a wide range of strategic and operational decisions, there are no specific bodies charged with identifying strategic research priorities in the face of resource scarcity. To claim legitimacy, such a body would have to be independent, with a mandate to advise research funders and funding consortia on an entire field of research, such as microbicides, or perhaps even more broadly, such as across HIV prevention approaches. This body would review the relevant science to make periodic assessments of current technologies against the most promising new alternatives, would be established external to the existing trials, and would have representation from all legitimate stakeholders. Most importantly, it would provide recommendations about stopping established trials for opportunity costs when it felt there was a compelling scientific rationale to do so.

One framework that could be used for priority setting is known as “accountability for reasonableness,” a framework that is grounded in justice theories emphasizing democratic deliberation [13]–[18]. This framework requires four conditions to be met for fair priority setting.

*Relevance* would require sound arguments supported by credible evidence about why an alternative strategy would be superior and a conclusion that stopping the current trials and pursuing alternative strategies is the most responsible way to use the available resources. *Publicity* would require that individuals and communities who might agree to participate in clinical trials be made aware of the proposed advisory body and its mandate as a routine part of informed consent. The “opportunity costs approach” must be transparent, i.e., the rationales for stopping would have to be publicly accessible and open to scrutiny. The *revision* condition would require that those who might disagree with recommendations of the advisory body be given an opportunity to present new evidence or arguments. This condition would require a very clear and efficient process. Finally, *enforcement* would require the sponsoring organization(s) to be accountable for ensuring these foregoing conditions are met [14].

### How Would Early Stopping Decisions for Opportunity Costs Be Implemented?

We envision two possible mechanisms for implementing early stopping for opportunity costs—either creating a new type of scientific oversight committee or simply expanding the mandate of existing DSMBs. Mechanisms such as those we propose here were explicitly advocated by the *Nature* editorial cited above [1]—and may also have broader applicability in global health research.

#### Option 1: The scientific oversight committee

An independent advisory board—a scientific oversight committee (SOC)—could be established by the research partners in much the same way that DSMBs are currently created [19]. The SOC's mandate would be to review all relevant science in the field and to make periodic assessments of the performance of the research against the most promising alternatives. The legitimacy of the SOC would be derived from the agreement and representation of the principal parties involved, all of whom have a stake in the conduct and outcome of the trial: funders, researchers, communities engaged in the research, DSMBs, relevant institutional review boards, and possibly the relevant regulatory authorities. An oversight process would need to be established, with a broad remit that takes into account these varied interests before a decision is made to stop any trials early.

The scope of the SOC's mandate would depend on the degree of cooperation and coordination among research funders and the degree to which the analyses and recommendations of the SOC were perceived to be well-founded, independent, and fair by the broad community of stakeholders. The SOC process could also help funders in collective decision-making about jointly supported clinical trials.

**Figure 1:**
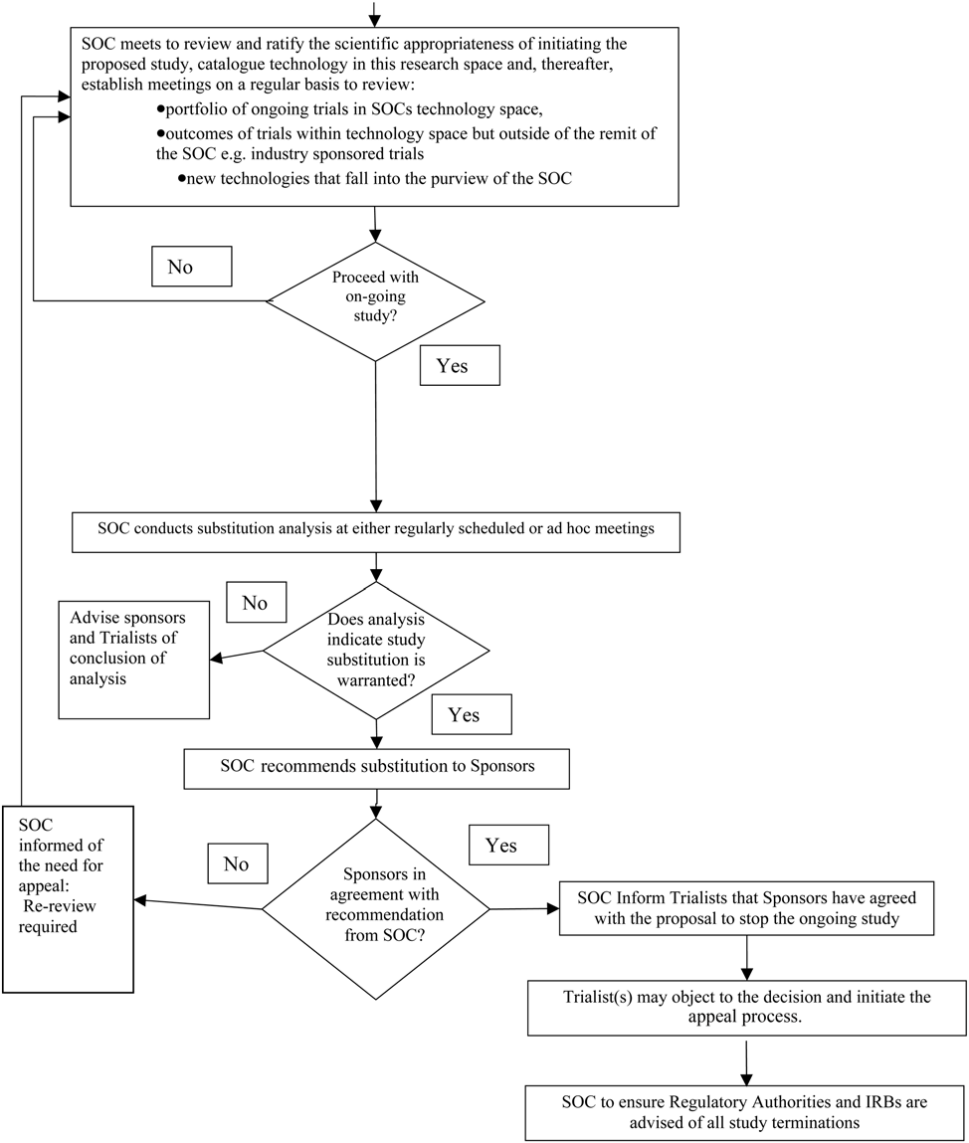
The proposed SOC decision-making process.

The SOC would be required to consider factors outside the current trial in determining whether stopping an existing clinical trial and investing instead in a new trial is warranted. It would be essential that the SOC work closely with the DSMBs and investigators throughout the relevant trials. Information from the ongoing studies and updates of the most recent scientific and clinical data would need to be shared throughout. The proposed SOC decision-making process is illustrated in Figure 1.

The SOC represents a new type of committee and therefore is likely to encounter some initial resistance in an already complex and bureaucratic field. Although we envision the SOC as having an advisory mandate, in order to satisfy the conditions of accountability for reasonableness, it would have to deal especially with strong differences of opinion among stakeholders and appeals for revisions to SOC recommendations. A key challenge in this regard would be to design a process that could reliably and efficiently share the necessary information, and accommodate new rationales, while avoiding becoming a platform for the personal commitments of champions of one specific product or another. One advantage of an SOC is that it might assist funders to work together in a particular area—for example, if a large foundation and a public research funding agency struck a joint SOC.

#### Option 2: Expanding the mandate of existing DSMBs

An alternative to the creation of the SOC, but one designed to accomplish the same goals, would be to expand the mandate of existing DSMBs. These boards already take external rationales into account in their decisions to recommend stopping trials early, though they lack the necessary frameworks and procedures for doing so in ways that might be viewed as transparent and fair [12]. Option 2 would require networking of DSMBs in a field of research, and also supplementing DSMB membership with representatives of participating research communities, for reasons of legitimacy discussed above. The advantage of building upon DSMBs is that the existence, independence, acceptability, and accountability of DSMBs is already widely recognized and accepted. Likely challenges for this approach would include overcoming resistance to changes in the DSMB mandate, and to decision-making standards that would involve reasoned judgments about a broader range of evidence than is currently reflected in statistical early stopping rules employed by DSMBs [20].

### Conclusion

We must ensure that we are getting promising technologies in the hands of people who need them as quickly and efficiently as possible. A current inefficiency in clinical trials, as highlighted by the microbicide case, is the inability to reallocate clinical trial resources to scientifically more promising technologies. We have proposed a solution—stopping clinical trials early on the basis of opportunity costs in global public health, following clear standards and careful and fair processes. The cost of inefficiency in clinical trials in global health is measured in lives of the poor. Under these circumstances, the status quo is not an answer.

## David Buchanan's Viewpoint: Stopping Trials Early for Opportunity Costs Is Practically Infeasible and Ethically Indefensible

James Lavery and colleagues recommend a new standard for stopping clinical trials early, based on “opportunity costs,” the potential waste of limited resources for completing trials currently underway while testing of potentially “more promising technologies” is delayed. They propose that a body be charged with responsibility for monitoring scientific developments. If more promising technologies emerge, then this body should be authorized to stop current trials in order to reallocate the financial, material, and human resources from the ongoing study to start a clinical trial of the new intervention expeditiously. Since criteria for assessing which technologies may be more promising are “inherently uncertain,” they recommend that this body use a fair procedural process, based on Daniels and Sabin's accountability for reasonableness model, to decide when to stop a trial for opportunity costs. Their proposal, however, is ethically indefensible and practically unworkable.

### Potential Harm to Participants Cannot Be Justified

The ethically fatal flaw in Lavery and colleagues' proposal is that they fail to address how the potential harm to the participants in the trial being considered for early stopping can be justified. Because research inherently poses the threat of harm, the risk of enrolling participants in clinical studies must be counter-balanced by the benefit of generating new knowledge. Since stopping a trial early severely limits the knowledge gained by a study, there must be an overriding ethical reason, like preventing harm, to justify early stopping.

DSMBs may legitimately stop trials early for three reasons: (1) harm (the rate of serious adverse events is higher in the experimental arm than in the comparison group); (2) efficacy (the rate of the desired outcome in the experimental arm is significantly higher than expected, based on the hypothesized effect size); and (3) futility (emerging effects are much smaller than hypothesized, indicating that it will be impossible to draw definitive conclusions if the trial is continued, based on the sample size for which the trial was powered). A fourth reason, technically not “early stopping” but “unblinding,” is based on the emergence of evidence of the superior efficacy of an intervention in an independent trial, which triggers a recalibration of the standard of care in concurrent trials.

The ethical rationale for stopping trials early for efficacy or for unblinding studies is based on the harm caused by depriving participants of a more effective treatment. Continuing the current trial is no longer ethically justifiable because treating research volunteers with a therapy now known to be inferior would result in higher levels of morbidity and mortality than providing the new, more effective therapy. Lavery and colleagues' proposal for stopping trials early for opportunity costs seems to resemble the reasoning behind decisions to stop trials early for efficacy or unblinding, but with crucial differences.

First, the risk to participants could no longer be justified because little-to-no significant knowledge would be gained from the trial stopped early. In agreeing to be randomly assigned, the participants were willing to expose themselves to the threat of harm for the sake of advancing science, but this counter-balancing benefit would be voided if the proposed “opportunity cost” stopping rule was invoked. The critical difference with the new proposal is that, unlike early stopping for efficacy or unblinding, parties to this proposal could not claim that the participants were being exposed to a treatment that is now known to be inferior or recommend a new therapy known to be superior. Thus, exposure to the inherent risk of research could not be justified by the new knowledge to be gained (since virtually none will), nor could researchers claim that the trial must be stopped based on the overriding moral imperative to prevent harm by providing access to a known better treatment (since none is available).

Furthermore, for participants assigned to the experimental condition, another ethically significant difference between the Lavery proposal and established warrants for early stopping is evident. In cases of unblinding, researchers can gain valuable knowledge by comparing study participants who choose to switch to the new “standard of care” with those who decide to continue with the original experimental treatment. Continuing the trial is justified because it will provide researchers and participants with greater knowledge about the effects of the original experimental intervention, even under these less than ideal conditions; the trial can proceed based on the warranted assumption that the original intervention could still prove to be efficacious and possibly superior to the newly established standard of care.

Unlike cases of unblinding, however, the option of continuing the trial of the “older” experimental therapy would be foreclosed under the early stopping for opportunity costs proposal, as it would contradict the intent to reallocate resources to a new trial. Without the clear need to stop the trial to prevent harm, participants in the treatment arm would thus be put in the ethically indefensible situation of having been exposed to an experimental intervention where its potential benefit or harm will be left undetermined. Significantly, as the early stopping of several recent microbicide trials shows, research inevitably entails risk and, despite researchers' best efforts, experimental interventions sometimes cause harm. The proposed new standard is ethically unacceptable because participants in trials stopped for opportunity costs would never know whether they were benefited or harmed by the original experiment, nor would this potential harm be mitigated by the provision of a better alternative.

Finally, the authors are vague about who they see as being harmed. Passing references to public health goals suggest that they believe stopping trials early is justified based on the potential benefit to the population as a whole, implying that this population is being deprived of the benefits of the more promising technology. If this is their intent, then the authors would be shifting grounds from the harm to the research participants to the potential harm to society. The authors may wish to stake their claim here, but if so, they need to make the case explicit and address well-known criticisms of utilitarian arguments that harms to the individual (enrolled in the trial) are justified on the grounds of the greater good to society.

### The Proposed Decision-Making Process Is Impractical

The proposal is also untenable from a practical standpoint. Currently, early stopping rules are based on strict extrapolations of statistical conventions for ruling out type I errors (erroneously concluding a difference exists when there is none). While everyone agrees that the standard *p*<0.05 is arbitrary, it is an accepted scientific convention that has served the advance of scientific progress well. Unlike stopping guidelines now used by DSMBs, which are based on the degree of scientific certainty, Lavery and colleagues propose that this criterion should be replaced by a procedural standard, based on Daniels and Sabin's accountability for reasonableness model.

Daniels and Sabin developed their model for use in deciding which services health insurance plans should cover [15]. A board is charged with fiduciary responsibility for deciding, prototypically, whether the insurance pool should cover one person's enormous expenses for a rare yet horrendous disease, versus providing dental care for everyone enrolled in the plan. Daniels and Sabin argue that their procedural model is necessary because the different values at stake do not have a common metric, and hence, the only fair way to decide how to allocate limited resources is to use fair procedures, such as a majority vote by the board, about which services the plan should cover.

The authors recommend that the body charged with stopping trials early for opportunity costs use the same process. In contrast to DSMB decisions based on scientific certainty, Lavery and colleagues' proposal would open deliberations to a virtually unlimited range of value considerations regarding the promise of new technologies. For example, “promise” could be measured on the basis of potential efficacy, seriousness of the health problem, size and characteristics of populations affected, prevention versus treatment, and so on [21]. To press the point, many people argue that promoting gender equity would be more effective in preventing HIV/AIDS than developing new medical interventions. In such situations, board members would be faced with weighing considerations ranging from evidence from epidemiological studies versus animal models, the relative importance of primary versus secondary outcomes, reducing health disparities versus developing universal interventions, short-term versus long-term results, and so forth. Since these different values cannot be put on a single scale, the authors propose that this body must use a fair procedure, such as majority vote, to make the decision to stop a trial early in order to divert the resources to the more promising intervention.

The most immediate problem with this proposal is that the authors state that their proposal must include an appeals process, but it is difficult to imagine that disputed decisions will be resolved quickly. This is particularly the case if one assumes that the premise of the authors' argument is true, i.e., that reasonable people can reasonably disagree about which technology is more promising, hence the need for resorting to a procedural process to make the decision. Researchers and sponsors who stand to lose would invariably seek to introduce new evidence to make their case that they should be allowed to proceed. It is difficult to imagine, for example, that Merck will quietly stand by while this body diverts public resources from research on its product to support research on a rival product developed by Pfizer; or that Anthony Fauci will accept that this body can stop his research because they think that Robert Gallo's research is more promising, particularly when these decisions could, in principle, be made on the basis of a split eight-to-six vote. The most likely result is that the appeals process would take years, which would ultimately defeat the purpose of moving money and resources into a new investigation quickly.

Ultimately, the proposal is impractical because it does not provide sufficiently clear standards to generate the trust and confidence necessary for acceptance and buy-in. This concern stems from the related problems of failing to define how representation on the decision-making body will be determined and failing to provide more substantive criteria for their decision-making. The authors state that all “legitimate stakeholders” must be represented on the body, but provide no guidance on the number or definition of stakeholders who should be considered legitimate. They then expect that those parties who would be most directly impacted by the decision will agree in advance to the proposed new early stopping rule. But it is not clear why the invested parties would accept the proposed new standard, given the uncertain terms for decision-making. Ultimately, the credibility of any such body would be untenable because they are not accountable to anyone; without true fiduciary responsibility for a defined set of resources, they will not have the trust or authority to adjudicate among the competing interests.

### Conclusion

The opportunity costs proposal would create distinct conditions previously unaddressed in analyses of early stopping practices. Under the conditions proposed by Lavery and colleagues, participants would be asked to enroll in a trial with the possibility that the threat of harm would not be counter-balanced by the benefits of generating new knowledge, even when there are insufficient scientific grounds to advise participants to start a new alternate therapy known to be superior. In addition, participants in the treatment arm would be exposed to an experimental intervention that inherently posed the threat of harm, and then left stranded without knowing whether they were harmed or not. The authors claim that it will be ethically sufficient merely to forewarn potential participants about these possibilities in the informed consent document, but they offer no indication about how the potential harms discussed here can be justified. Finally, because decisions about early stopping for opportunity costs would invariably provoke heated disputes, precisely because there would be no accepted substantive standards but ultimately only a procedural vote, the proposal fails to provide compelling grounds for recognizing the board's authority for making such consequential decisions.

## James Lavery and Colleagues' Response to David Buchanan

The overarching theme of David Buchanan's response to our proposal is to reject it because it is new—it would, he says, “create distinct conditions previously unaddressed in analyses of early stopping practices.” Ultimately, the global health community and the patients we serve will have to judge whether loyalty to traditional decision-making conventions in clinical trials has helped bring poor women at risk of HIV infection and other diseases the effective preventions and treatments they deserve, with the urgency the problem demands.

In our proposal, we stated that “up-front commitments must be made to ensure that any early stopping of a trial does not diminish the standard of care provided to individuals enrolled under the initial enrollment agreement.” Buchanan rejects this approach. He argues that stopping a clinical trial early for opportunity costs would harm research participants beyond standard of care concerns, apparently by thwarting their important interests in contributing to the advancement of science, which he views as a “counter-balancing benefit” for assuming the risks of participation. He does not acknowledge that those interests might be *better* served by adopting our proposal, rather than locking participants into a trial of a potentially inferior product.

Buchanan contrasts the decision-making process that we outline in our proposal with “DSMB decisions based on scientific certainty.” His worry appears to be that the level of “certainty” and authority of DSMB decision-making for a particular trial would be subverted by an endless range of “value considerations,” including the “seriousness of the health problem, size and characteristics of populations affected, prevention versus treatment, and so on.” In fact, DSMB decision-making procedures rarely, if ever, approach certainty, and the various “value considerations” Buchanan describes are frequently reflected in DSMB deliberations, even if they are not formalized into decision-making algorithms. Our original proposal argued that the “substantive standard for decisions to stop an existing trial in favor of a strategic move to another product should be that a trial of the new product is ready to begin and that there is a compelling scientific rationale for why the new intervention may be considered more promising.” We do not propose substituting DSMB procedures with ideological debates about health and development more broadly, as Buchanan suggests.

We were first prompted to develop our proposal by widely reported negative trials in the field of microbicides. Recently, the PRO 2000 gel phase IIb trial reported a trend towards efficacy, the first microbicide trial to do so [22]. Further information about the efficacy of PRO 2000 is expected later this year when the results of the larger UK Medical Research Council–funded MDP 301 trial are announced.

Millions of women have been infected with HIV since research on microbicides began in the early 1990s, and since the epidemic continues relentlessly, we must do everything in our power to maximize the efficiency with which the field of HIV prevention gains answers to important clinical questions.

## David Buchanan's Response to Lavery and Colleagues

To be clear, I am seriously concerned that Lavery and colleagues' proposal would create new conditions that pose new and unprecedented risks to trial participants. The novelty of their proposal is that they maintain that the principle of non-maleficence should be discounted. I reject this proposition. It is a misguided and treacherous position for Western bioethicists to be taking about the conduct of trials in international settings.

In response to my critique, Lavery and colleagues suggest that they have not broadened the purview of their proposed new board beyond that of the deliberations now standard for DSMBs. If this is the case, then it is not clear why they have proposed the creation of a new body and one that they recommend use a different set of decision-making rules, defined by the accountability for reasonableness model. This model was conceived precisely to handle situations in which there are distinct and conflicting value considerations at stake, conflicts for which the only fair resolution is to shift to a procedural mechanism for resolving such disputes (such as a majority vote). If Lavery and colleagues truly believe that their proposal can be implemented under the tightly bounded criteria of scientific certainty that DSMBs now use, then it is not clear why they have proposed abandoning that standard and replacing it with decision-making rules that were created for situations with inherent value conflicts.

Their stance that we must maximize efficiency over all other considerations, and in particular, over concerns for the safety of research participants, is as stunning as it is unacceptable.

## Abbreviations

DSMB: data safety and monitoring board
SOC: scientific oversight committee
